# Assessment of 9-OH- and 7,8-diol-benzo[a]pyrene in Blood as Potent Markers of Cognitive Impairment Related to benzo[a]pyrene Exposure: An Animal Model Study

**DOI:** 10.3390/toxics9030050

**Published:** 2021-03-08

**Authors:** Lynda Saber Cherif, Lei Cao-Lei, Sophie Farinelle, Claude P. Muller, Jonathan D. Turner, Henri Schroeder, Nathalie Grova

**Affiliations:** 1 Calbinotox, EA7488, Faculty of Science and Technology, Lorraine University, 54500 Vandoeuvre-lès Nancy, France; lynda.saber-cherif@univ-lorraine.fr (L.S.C.); lei.cao@mail.mcgill.ca (L.C.-L.); henri.schroeder@univ-lorraine.fr (H.S.); 2 Immune Endocrine Epigenetics Research Group, Department of Infection and Immunity, LuxembourgInstitute of Health, L-4354 Esch-sur-Alzette, Luxembourg; jonathan.turner@lih.lu; 3 Experimental & Molecular Immunology Research Group, Department of Infection and Immunity, Luxembourg Institute of Health, L-4354 Esch-sur-Alzette, Luxembourg; Sophie.farinelle@lih.lu; 4 Department of Infection and Immunity, Luxembourg Institute of Health, L-4354 Esch-Sur-Alzette, Luxembourg; claude.muller@lih.lu; 5 Laboratoire National de Santé, L-3583 Dudelange, Luxembourg

**Keywords:** benzo[a]pyrene, oral exposure, 9-OHbenzo[a]pyrene 7,8-diol-benzo[a]pyrene, biomarker of neurotoxicity, cognitive impairments, NMDA receptor, metabolism

## Abstract

The potent neurotoxicity of benzo[a]pyrene (B[a]P) has been suggested to be a susceptibility factor accelerating the onset of brain tumours and the emergence of neurobehavioural disturbances. B[a]P has been shown to be neurotoxic, acting directly on both the central and peripheral nervous systems, as well as indirectly via peripheral organs like liver and gut. By using a realistic B[a]P exposure scenario (0.02–200 mg/kg/day, 10 days) in mice, we elucidated brain-specific B[a]P metabolism and at identified hydroxylated B[a]P metabolites in serum which could be used as markers of cognitive impairment. Repeated oral administration of B[a]P led to, at the doses of 20 and 200 mg/kg/day, significant overexpression of Cyp1a1/Cyp1b1 in 2 out of the 3 brain regions considered, thereby suggesting the ability of the brain to metabolize B[a]P itself. At the same doses, mice exhibited a reduction in anxiety in both the elevated plus maze and the hole board apparatus. Concomitantly, B[a]P triggered dose-dependent changes in *Nmda* subunit expression (Nr1 and Nr2a/Nr2b) in areas involved in cognition. We detected 9-OH-B[a]P and 7,8-diol-B[a]P in serum at the level for which cognitive impairment was observed. We suggest that these metabolites may, in the future be exploited as potent biomarkers of B[a]P-induced cognitive impairments.

## 1. Introduction

A limited set of data suggests the potent neurotoxicity of Polycyclic Aromatic Hydrocarbons (PAHs) as a susceptibility factor for both the onset of brain tumours in adults [1] and children [2] and the emergence of neurobehavioural disturbances during development and adulthood [3,4]. Among PAHs, benzo[a]pyrene (B[a]P) is proven to be neurotoxic, acting directly on both the central (CNS) [5,6,7] and peripheral nervous systems [8], as well as indirectly via peripheral organs like liver and/or gut [9]. In the latter case, abnormal peripheral function triggers abnormal brain activities (e.g., hepatic encephalopathy) [10]. Epidemiological data also suggests that occupational B[a]P exposure is linked with the non-carcinogenic neurotoxic effects of the latter. In Poland, the prevalence of various short-term-memory disorders was demonstrably higher with increased B[a]P exposure levels in coking plant workers [11]. Similarly, coke-oven workers in China had lower learning-ability and memorization-test performance that correlated with levels of the PAH 1-OH-pyrene in urine [12]. Mechanistically, exposure to B[a]P was linked to memory and learning disabilities in these coke-oven workers by a reduction in choline, monoamine and amino acid neurotransmitter levels [12,13]. Occupational exposure is not the only concern, however. Populations living in proximity to PAH-emitting plants such as those recycling engine-oil and chemical-waste (Baton Rogue, LA, USA) have also been linked to neurophysiological disorders such as depression, mental confusion or fatigue [14]. A similar relationship has been reported in the population residing near a creosote-production plant in Mississippi (USA) [15].

The brain appears to be specifically targeted by PAHs including B[a]P by reason of their strong lipophilicity and accumulation in lipid-rich tissues [16,17]. In addition to the ability of CNS tissues to metabolize these organic-pollutant compounds in both their free and plasma lipoprotein bound states can pass the blood–brain barrier (BBB) [18], increasing the pool of PAHs and their potentially reactive metabolites in the brain [5,17,19]. We have previously demonstrated that repeated intraperitoneal exposure to B[a]P over periods as short as 10 days, induced behavioural abnormalities including anxiety, short-term memory and motor activity losses [5,6] in a dose-dependent manner. These behavioural changes correlated with oxidative stress levels and to alterations in the N-methyl-D-aspartate (NMDA) *Nr1* and *Nr2a* subunit mRNA expression in the hippocampus as well as frontal and temporal cortexes [5,6,20]. The NMDA receptor is an ionotropic heterotetrameric receptor, highly variable, consisting of two NR1 and 2 NR2 subunits that delineate the central Ca2+ permeable channel [21]. There are several genes encoding the NR1, NR2A-D, NR3A or B subunits considerable increasing the complexity and possibilities for the constitutive clustering of the receptor. The widely distributed NR1 subunit is of crucial importance for the functioning of the receptor. The NR2A and NR2B subunits have a unique spatial expression pattern, preferentially expressed in the amygdala, hippocampus and frontal cortex with the modulation of the functioning of the receptor and the intracellular Ca2+ homeostasis as a physiological role. For instance, receptors containing NR2A subunits present higher signal-transmission speed than those constituted with NR2B. NMDA receptor functionality and plasticity is known to be heavily impacted by exposure to environmental chemicals, especially PAHs [22]. Furthermore, increased *Nr2b* promoter DNA methylation prevented B[a]P-induced learning impairments and was linked with the downregulation of *Nr2b* mRNA expression [23,24]. Other PAHs like fluorene or fluoranthene were also demonstrated to induce similar behavioural disturbances according to the dose administered, corroborating the potent neurotoxic effects of these environmental chemicals [25,26]. Altogether, these studies further highlighted the strong correlation between the intensity of the behavioural effects and the cerebral concentration of PAHs as well as their metabolites. The accumulation of the metabolites in the brain suggests that the metabolism of PAHs may somewhat increase their neurotoxicity [6,25,26]. Exploring brain-specific B[a]P metabolism following a *per os* administration and the relationships between brain and blood compartments are aimed at: (i) having better knowledge about the roles that the entero-hepatic and brain metabolism play in the neurotoxicity of B[a]P exposure; and (ii) identifying specific metabolites in serum which could be indicative markers of cognitive impairments triggered by B[a]P exposure. The present study has therefore been designed to evaluate the brain-specific metabolism of repeated oral B[a]P-administration in mice which were concomitantly assessed for their behavioural abilities and regional glutamate *Nmda* receptor subunits expression in three targeted brain regions.

## 2. Materials and Methods

### 2.1. Animals

Balb/c mice (18–20 g, females *n* = 60, Charles River Laboratories, Ecully, France) were kept in open polysulfone resin cages. Animals had a 1-week acclimatisation period and were maintained under reversed light/dark cycles (red light from 7:00 a.m.; white light from 7.00 p.m.) at 22 °C ± 2 °C and 55 ± 10% relative humidity) and had access to food and water ad libitum. B[a]P (>97% purity, Sigma Aldrich, Bornem, Belgium) and solubilized in ISIO 4 vegetable oil (Lesieur, Neuilly sur-Seine, France). Animals were randomly allocated by 10 to each of the experimental groups and received either 0.02, 0.2, 2.0, 20 or 200 mg/kg/day of B[a]P in vegetable oil. B[a]P was diluted to an administration volume of 5 mL/kg b.w and administered by oral gavage for 11 consecutive days. Control animals received the vehicle only. The lowest B[a]P doses, ranging between 0.02 and 2 mg/kg, were representative of levels observed in smokers, occupational exposure or heavy consumers of grilled or smoked foodstuffs [27,28,29]; whereas those from 20 to 200 mg/kg were often considered as B[a]P toxic range [29].

Anxiety, physical- and learning-activity related behaviours were assessed 60 min after the administration of B[a]P on day 10. On day 11, mice were sacrificed 180 min after the last B[a]P dose, blood collected, and 3 brain regions were dissected from the brain (temporal cortex, frontal cortex and hippocampus) using the method of *Glowinski and Iversen*. All biological specimens were stored at −80 °C until subsequent use.

### 2.2. Serum and Brain Concentration of B[a]P and Hydroxylated B[a]P Metabolites

At the end of the exposure period, extraction and purification of all analytes from serum and brain were carried out as previously described [6]. Analysis of B[a]P and its hydroxylated forms was then performed by high performance liquid chromatography (HPLC), and detection was by fluorescence (Agilent 1100 Series, Machelen Belgium). Specimen (500 µL) was loaded on a reversed-phase Vydac C18 guard column (250 mm × 4.6, I.D., 5 µm particle size, VWR, Leuven, Belgium) and the elution of the analytes was carried out as follows: (i) an initial 5 min isocratic elution with acetonitrile-water-trifluoacetic acid (20:79.9:0.1; *v*/*v*/*v*) at 1.5 mL/min, (ii) a second 5–10 min linear gradient from 20% up to 60% acetonitrile, (iii) a third 10–20 min with a linear gradient up to 100%, (iv) a fourth 20–22 min 100% acetonitrile, (v) a fifth 22–24 min with linear gradient from 100% to 20% acetonitrile and vi) finally 24–30 min with 20% acetonitrile. The identification and quantification of B[a]P and its hydroxylated-metabolites were made by the comparison of peak areas and retention times to standard solutions (MRI/NCI, Kansas City, MO, USA). Fluorescent detection was performed using excitation and emission wavelengths of 380–430 nm for B[a]P, its 1-OH-, 3-OH-, and 9-OH metabolites while 7,8 diol-B[a]P was measured at 370–410 nm. The quantification limits were determined between 0.12 and 2.6 ng/mL in serum, and 0.12–1.2 pg/mg in brain, depending on the analyte (validation methods details in [6]). The percentages of recovery were determined by using the total amounts of B[a]P, sum of OH-B[a]Ps and 7,8 diol-B[a]P expressed in molar percentages.

### 2.3. Nmda and Cytochrome Gene Expression

Frontal cortex, temporal cortex and hippocampus were grinded under powder based on a cold-milling process to homogenized tissues. Purification of mRNA was then performed on 10 mg of powder by the means of a μMACSTM mRNA isolation kit (Miltenyi Biotec, Leiden, The Netherlands). Superscript II (Thermo Fisher Scientific, Merelbeke, Belgium) was used for first-strand cDNA synthesis as previously described [30]. Quantitative PCR amplification was carried out in an Opticon 2 (MJ Research, Bio-Rad, Nazareth, Belgium) using Platinum Taq DNA polymerase (Thermo Fisher Scientific, Merelbeke, Belgium). Primers and PCR conditions were as previously described for *NMDA* [6] and *Cyp1a1/1b1* [30]. We have previously confirmed the stability of *Gapdh as* reference gene in brain [31]. RT-qPCR results for *Nmda* subunits (-R1, -R2A and -R2B) were as gene copies/10^2^ *Gapdh* copies. Regardless of accurate fragment size, the limit of detection was established at 38 cycles. For the cytochromes *Cyp1a1* and *Cyp1b1*, the gene expression was normalized to those of *Gapdh/Ppia* and expressed using the 2-ΔΔ^Ct^ method [32].

### 2.4. Behavioural Testing

As animals were maintained under an inverse day/night schedule, behavioural testing was performed from 8.00 a.m. onwards under standard dim red light. Each test was recorded, and videotapes were subsequently trained observers blinded to the B[a]P exposure scored behaviour using The Observer XT (Noldus, Wageningen, The Nederlands).

Mice were assessed for anxiety and activity in the elevated plus maze [33] and the hole board apparatus [34] which are tasks of good performances strongly related to the integrity of brain areas (e.g., hippocampus, amygdala, frontal part of the cortex) [35]. In the same way, the Y maze and the Morris water maze are well recognized tasks for the assessment of spatial learning and memory performances [36]. Memory performances in such tests are highly related to synaptic plasticity and NMDA receptor functioning, especially in the hippocampus and interconnected brain site like entorhinal cortex [37].

Elevated-plus maze (EPM). The EPM was maintained 30 cm above the floor and consisted of four alternating open/closed arms (all 25 × 5 × 20 cm). Side-walls on closed arms were 20 cm high. Individual mice were permitted free access to all four arms for 5 min after being introduced into the central area of the maze, all facing the same open arm. Using the four-paw criteria, animals were scored on the frequency of closed- and open-arm entries as well as the time in the open or closed sections of the maze [38].

Hole board apparatus. An arena with 16 equidistant 3.5 cm internal diameter holes (4 rows of 4 holes) was held one meter above the floor. Individual mice were placed in the centre of the arena and behaviour recorded for 5 min. The arena was divided into 3 analysis areas, central holes, peripheral holes and the area outside the maze. Head dips (both eyes disappeared into the hole [39]) were scored for each of the three analysis areas. Head-dipping behaviour in the hole-board apparatus is an indicator of the exploratory behaviour and anxiety levels of mice while in the arena. To avoid interactions between the hole-board and the EPM, half of the mice underwent the hole-board first and then the EPM, and the remaining half the inverse order. Test order had no effect on the outcome, consequently all animals were included in the final dataset.

Y-maze. The maze consisted of three black-painted wooden arms (25 cm long, 5 cm wide, 14 cm high) radiating equiangularly from a central point. We assessed immediate working memory was assessed by placing individual animals at the end of one arm. Behaviour was recorded for 10 min. Observers counted spontaneous alternation between arms using the four-paw criteria for “successive entries into the three arms on overlapping triplet sets”[40]. Data were expressed as the percentage alternation. This was calculated as the actual/possible alternations ×100. The possible alterations were defined as the number of complete four-paw arm entries minus two.

Morris water maze (MWM). The MWM was a white, water filled (22 °C), opaque 60 cm diameter 75 cm deep plastic pool. An escape platform (10 cm × 10 cm) was maintained in a fixed location for all trials just below the water surface, hidden from view by powdered milk distributed over the water surface. An external visual clue was available. Mice were placed in the water diametrically opposite the cue, and the time necessary to reach and mount (30 s) the platform was recorded. Mice were tested in 5 consecutive trials (trial 1–5). Tests were considered a failure when the escape platform was not found within 3 min, animals were then retrieved and placed on the platform (30 s). The procedure was repeated for a single trial the following day without the escape platform. The time spent in the quarter of the maze previously containing the escape platform and the number of crossings of this zone were recorded. All trials were recorded, and escape latencies quantified by qualified observers.

### 2.5. Statistical Analysis

Normally distributed parametric gene expression data were analyzed by one-way ANOVA including B[a]P doses as an independent factor. Significant main effects (*p* < 0.05) underwent post hoc testing using the Student-Newman-Keuls (SNK) procedure.

For non-Gaussian-distributed data, non-parametric tests were performed using either a Kruskal–Wallis (KW) or Friedman test as appropriate. When the main effect was significant, either a modified Mann–Whitney procedure (MW) or a modified Wilcoxon test was applied, respectively. All statistical tests were performed using SPSS 11.5 (SPSS inc., Chicago, IL, USA). For all tests, differences were considered significant at *p* ≤ 0.05.

## 3. Results

### 3.1. Body Weight

Repeated administration of B[a]P during 11 day period did not affected body weight, irrespective of dose (Supplementary Figure S1). However, a small but non-significant decrease in body weight was monitored for the group administered with 200 mg/kg/day that was from a reduction in growth-rate during the last days of exposure (Supplementary Figure S1). It should been noted that the well-being of the animals in this group was considered satisfactory allowing us to complete the experiment.

### 3.2. Concentration Levels of B[a]P and Hydroxylated-Metabolites in Serum and Brain

**Figure 1:**
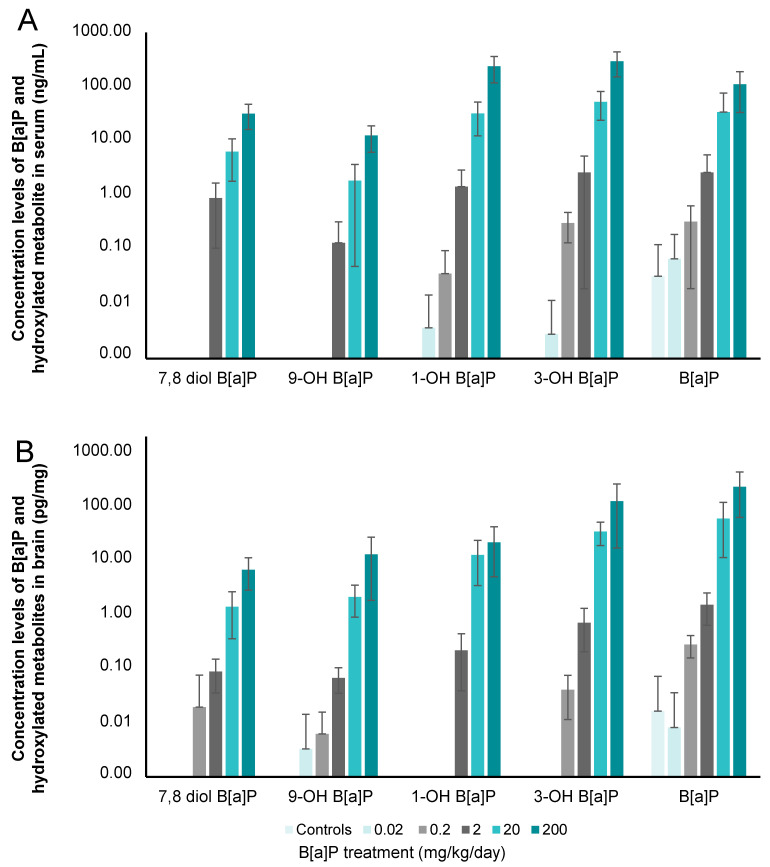
Concentration levels of B[a]P and hydroxylated metabolites in (**A**) serum and (**B**) brain in B[a]P-exposed mice *via* oral administration using doses up to 200 mg/kg for 11 consecutive days. Data are mean +/− SEM (*n* = 10 per group).

B[a]P has a low bioavailability for the organism. As expected, we detected only 0.04 to 0.08% of B[a]P and its metabolites in serum 180 min after the last oral administration. In the cortex however, this increased to between 0.97 and 3.52% of the total quantity of B[a]P administrated found as either the parent molecule or its metabolites. This confirmed the strong affinity and accumulation of B[a]P in lipid-rich tissues such as the brain [16,17]. Control rats, raised under monitored environmental conditions, had levels of B[a]P that were above the LOQ, representing the environmental exposure background of the mice via inhaled air, water and food (Figure 1A).

**Figure 2:**
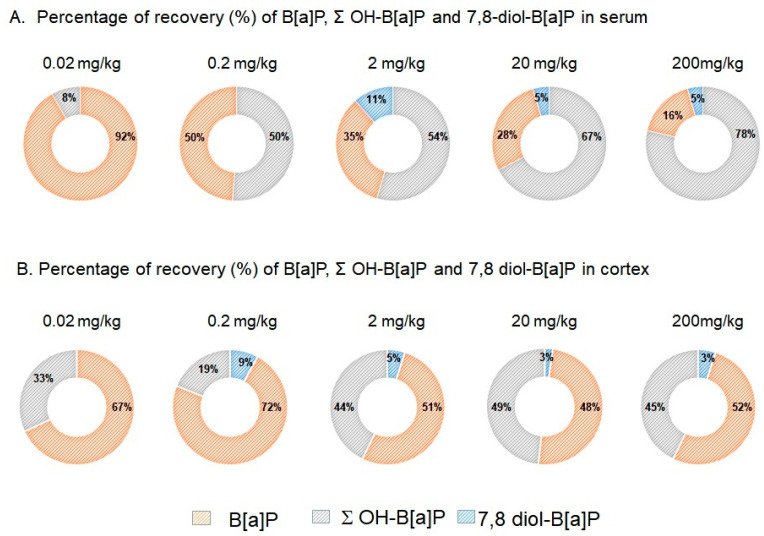
Molar percentage of B[a]P, sum of OH-B[a]P and 7,8-diol-B[a]P recovered in (**A**) serum and (**B**) cerebral cortex of mice exposed via oral administration using doses up to 200 mg/kg for 11 consecutive days. Data are mean +/− SEM (*n* = 10 per group).

Although B[a]P was detectable in the serum of control mice, 1-OH-B[a]P and 3-OH-B[a]P were detected in animals that received a minimum of 0.02 mg/kg/day B[a]P and 9-OH-B[a]P and 7,8-diol-B[a]P in those that received a minimum of 2 mg/kg/day B[a]P. 3-OH-B[a]P was therefore the most preponderant in serum, followed by 1-OH-B[a]P, B[a]P, 7,8-diol-B[a]P and 9-OH-B[a]P, irrespective of the dose administered (Figure 1A). The relative percentage of B[a]P in serum decreased from 92% at 0.02 mg/kg to 16% at 200 mg/kg in favour of its monohydroxylated forms for which an increase from 8% to 78% was observed at the same doses of exposure (Figure 2A). The reactive intermediate metabolite of B[a]P-diolepoxide, 7,8-diol-B[a]P, was detected in serum from 2 mg/kg upward. It represented 11% of the detectable B[a]P and metabolites at the dose of 2 mg/kg and at 5% at the two highest doses (20–200 mg/kg of B[a]P).

In the cortex, a different metabolite pattern was seen (Figure 1B). At 0.02 mg/kg, only B[a]P and 9-OH-B[a]P were detectable while all analytes except 1-OH-B[a]P were above the limit of quantification in mice exposed to doses of 0.2 mg/kg or higher. B[a]P was the most abundant in this matrix, followed by its 3-OH-B[a]P, 1-OH-B[a]P, 9-OH-B[a]P and 7,8-diol-B[a]P metabolites irrespective of the dose administered (Figure 2B). The relative percentage of B[a]P in cerebral cortex decreased from 100% in control group to 52% at 2 mg/kg. From the later dose to 200 mg/kg, equivalent proportions between B[a]P and metabolites were shown (Figure 2B). A repartition of 48%:52% was therefore observed at the highest dose (200 mg/kg) corresponding to the sum of mono- and dihydroxylated-B[a]P and B[a]P, respectively (Figure 2B). The 7,8-diol-B[a]P metabolite was measured in brain from 0.2 mg/kg upward and the relative level in the cerebral cortex decreased from 9% at 0.2 mg/kg to 3% at the two highest doses (2–200 mg/kg of B[a]P).

**Figure 3:**
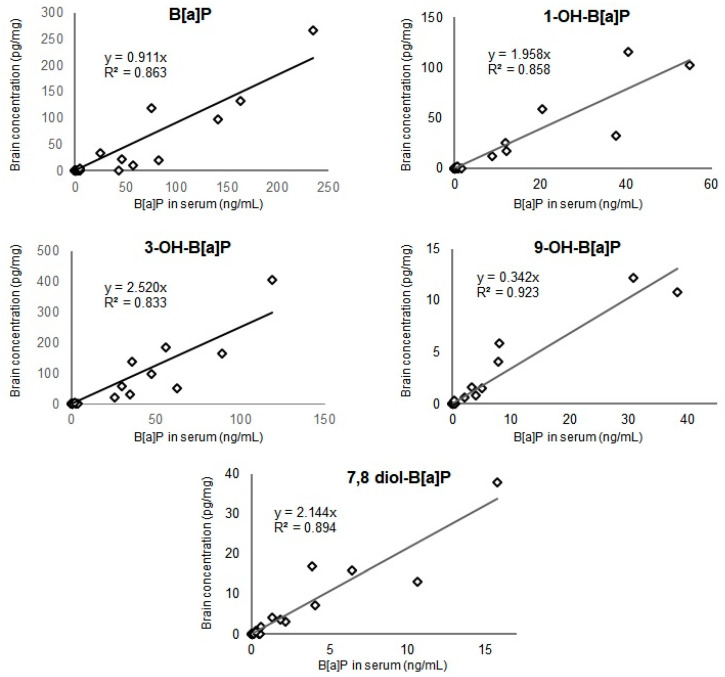
Linear relationship between the concentration levels of 7,8-diol-B[a]P, OH-B[a]P and B[a]P in serum and brain of mice exposed via oral administration using doses up to 200 mg/kg for 10 consecutive days (*n* = 28, *p* < 0.001, Spearman analysis).

There were strong linear relationships (R^2^ value comprised between 0.833 and 0.923 Spearman analysis, *n* = 28, *p* < 0.001) between the concentrations of B[a]P or OH-B[a]Ps in serum and their corresponding concentrations in the brain collected from exposed rats, as shown in Figure 3. The relationship between 9-OH-B[a]P in serum and brain was stronger (Figure 3, R^2^ = 0.923, *n* = 28, *p* < 0.001, Spearman analysis) followed by 7,8-diOH-B[a]P for which a R^2^ value of 0.894 was determined (*n* = 28, *p* < 0.001, Spearman analysis).

### 3.3. Expression of Cyp1a1 and Cyp1b1

**Figure 4:**
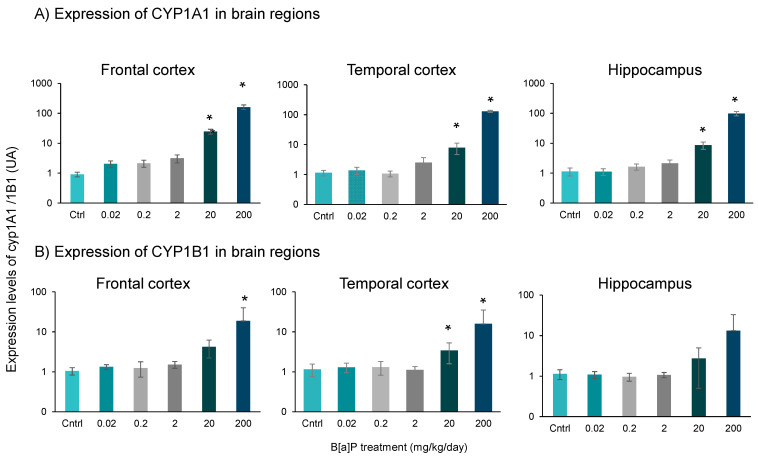
Levels of mRNA expression of Cyp1a1 (**A**) and Cyp1b1 (**B**) genes in brain regions (temporal and frontal cortex and hippocampus) in B[a]P-exposed mice *via* oral administration at doses ranging from 0.02 to 200 mg/kg for 11 consecutive days. Results were reported as average +/− SEM of 10 mice. * *p* < 0.05 vs. controls (SNK-t test for multiple comparisons).

Cytochromes P450 1A1 and 1B1 are known to play an important role in the oxidation of PAHs, including B[a]P [41]. *Cyp1a1* and *Cyp1b1* mRNA expression levels were therefore assessed in the frontal cortex, temporal cortex and hippocampus. Figure 4 displayed a significant increase in *Cyp1a1* mRNA expression (*p* < 0.01, SNK t-test for multiple comparisons) in all regions at the two highest doses of B[a]P (20 and 200 mg/kg) (Figure 4A). *Cyp1b1* mRNA expression was significantly increased in both frontal and temporal cortex at the dose of 200 mg/kg, and only in the temporal cortex at 20 mg/kg (Figure 4B). Although the *Cyp1b1* mRNA expression profile was similar in the hippocampus this did not reach statistical significance (Figure 4B). At the lowest doses of B[a]P (from 0.02 to 2 mg/kg), no significant changes of expression of *Cyp1a1* and *Cyp1b1* mRNA were showed.

### 3.4. Expression of Nmda Subunits

**Figure 5:**
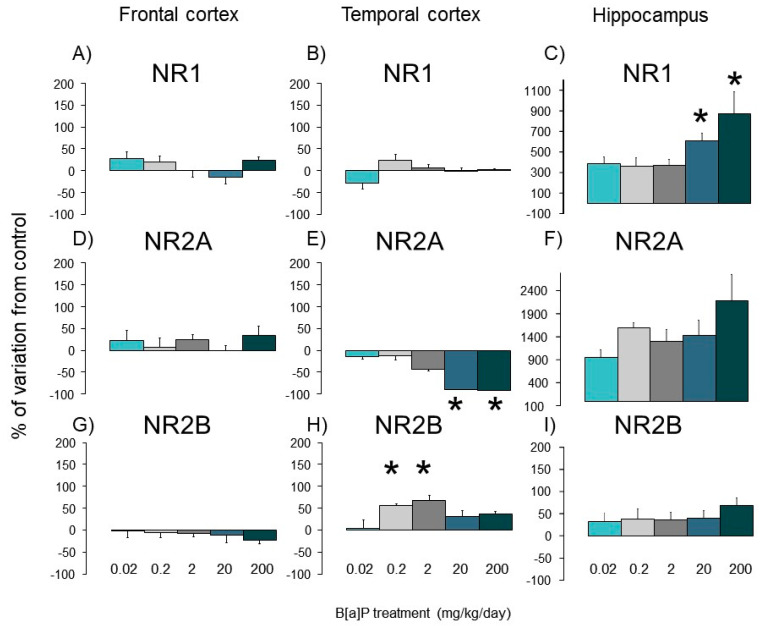
Levels of Nmda subunit (Nr1, Nr2a and Nr2b) expression in B[a]P-exposed mice via oral gavage at doses ranging from 0.02 to 200 mg/kg for 11 consecutive days. (**A**,**D**,**G**): respectively Nr1, Nr2a and Nr2b in frontal cortex. (**B**,**E**,**H**): respectively Nr1, Nr2a and Nr2b in Temporal cortex. (**C**,**F**,**I**): respectively Nr1, Nr2a and Nr2b in hippocampus. Nmda subunits are shown as copies/10^2^ copies Gapdh. Data were reported as mean ± SEM (4 mice per group). * *p* < 0.05 vs. controls (SNK-t test for multiple comparisons).

In controls, the mRNA levels of *Nmda-R1*, *-2A* and *-2B* varied greatly between the 3 brain regions investigated with 1300 copies of mRNA (per 10^2^ copies of *Gapdh*) in temporal cortex compared to only 39 and 50 copies for frontal cortex and hippocampus, respectively (Figure 5A,B). *Nr1 mRNA* is overexpressed significantly in the hippocampus of B[a]P-treated mice at the two highest doses (270 and 380 copies per 100 copies of *Gapdh*) compared to control animals (20 copies) (Figure 5C).

In contrast, B[a]P did not affect mRNA levels of the *Nr1* subunit in both temporal and frontal cortices irrespective of the dose (Figure 5A,B) and that of Nr2a expression in the frontal cortex and the hippocampus compared to controls (Figure 5D,F). In the temporal cortex, expression of the Nr2a subunit gene (mRNA) was downregulated in mice exposed to B[a]P at 2, 20 and 200 mg/kg although only the two highest doses reached statistical significance (Figure 5E, *p* < 0.05, SNK t-test for multiple comparisons). The temporal cortex was also the only part of the brain in which the mRNA of Nr2b subunit expression was dysregulated (*p* < 0.05, SNK t-test for multiple comparisons), increasing in mice treated with doses of 0.2 or 2 mg/kg, remaining similar to controls in the other B[a]P-exposed groups (Figure 5H).

### 3.5. Behavioural Effects of B[a}P

#### 3.5.1. Elevated Plus Maze

**Figure 6:**
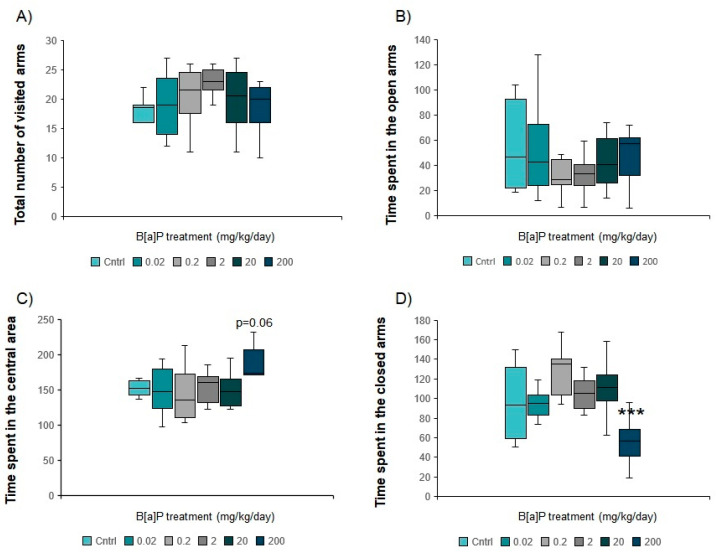
Evaluation of anxiety-related behaviour of B[a]P-exposed mice (oral gavage: 0.02 to 200 mg/kg, 10 consecutive days) in the EPM. (**A**): total number of visited arms, (**B**): Time (s) spent in the open arms, (**C**): Time (s) spent in the square central zone, (**D**): Time (s) spent in the closed arms. Data from 10 mice per group are expressed as the median (central line); boxplot represents the 1st (25%) and 3rd (75%) quartiles; minimum and maximum values observed are represented by error bars. *** *p* < 0.001 (MW-U test modified for multiple comparisons).

There was no significant difference in the total number of arms visited during the test, suggesting that B[a]P exposure did not affect the overall activity levels in this maze (Figure 6A).

Mice treated with B[a]P at 0.02 to 20 mg/kg spent equal times in all three parts of the maze (Figure 6B–D) which was not the case in those exposed to 200 mg/kg (*p* < 0.001, MW-U test modified for multiple comparisons). Although animals exposed to 200mg/kg spent a similar time in the open arms (Figure 6B), time in the central zone increased (*p* = 0.061, MW-U test modified for multiple comparisons, Figure 6C) with a concomitant reduction in time in the closed arms (*p* < 0.001, MW-U test modified for multiple comparisons, Figure 6D). The increase in time spent in the central area suggested that more time was needed to decide which zone of the maze to visit possibly related to a slight change in the level of anxiety. Indeed, this part of the maze is a zone of crucial importance for the decision-making and the initiation of the zone to be explored between the open and the closed ones [42].

#### 3.5.2. Hole Board Apparatus

**Figure 7:**
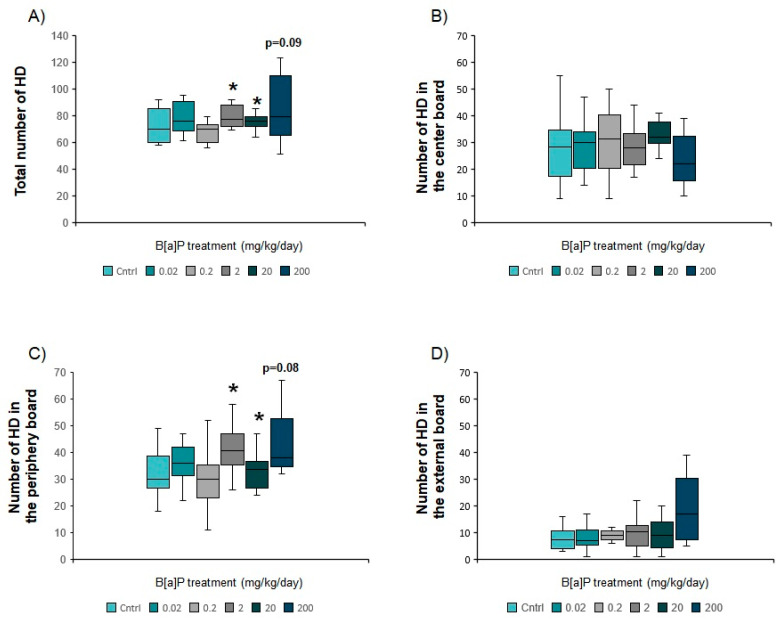
Anxiety-related behaviour of B[a]P-exposed mice via oral gavage at doses ranging from 0.02 to 200 mg/kg for 10 consecutive days in the hole board (HD) test. (**A**): Total number of holes, (**B**): number of HD in the central board, (**C**): Number of HD in the periphery board, (**D**): Number of HD in the external board. Data from 10 mice per group are expressed as the median (central line); boxplot represents the 1st (25%) and 3rd (75%) quartiles; minimum and maximum values observed are represented by error bars. * *p* < 0.05 (MW-U test for multiple comparisons). HD: head dipping.

There was a statistically significant increase in the total number of head dips relative to controls for mice exposed to B[a]P at 2, 20 and 200 mg/kg (12–26%, *p* < 0.05, MW-U test modified for multiple comparisons, Figure 7A). This suggests either an increased level of exploratory activity or a reduction in the level of anxiety. These animals also presented a significant and dose-dependent increase in the number of head dips in the peripheral area of the arena suggesting that dose- B[a]P reduced anxiety levels in a dose-dependent manner (Figure 7C). The non-significant increase in head dips off the edge of the apparatus in mice exposed to 200 mg/kg of B[a]P (Figure 7D) further supports this assumption.

#### 3.5.3. Y Maze

Irrespective of the B[a]P dose, no difference in the percentage of spontaneous alternations was observed (Supplementary Table S1). Similarly, B[a]P exposure did not affect any of the Y-maze activity measures (Supplementary Table S1).

#### 3.5.4. Morris Water Maze

**Figure 8:**
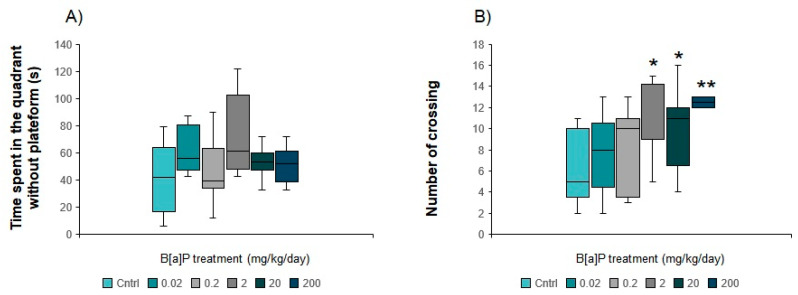
Performances of the sixth trial of B[a]P-exposed mice via oral gavage at doses ranging from 0.02 to 200 mg/kg for 10 consecutive days in the Morris water maze. (**A**): Time spent in the quadrant without plateform, (**B**): Number of crossing. Data from 10 mice per group are expressed as the median (central line); boxplot represents the 1st (25) and 3rd (75%) quartiles; minimum and maximum values observed are represented by error bars. * *p* < 0.05, ** *p* < 0.01, (MW-U test for multiple comparisons).

In all groups, the escape latency decreased significantly from trial 1 to trial 5 (Supplementary Table S2), showing that B[a]P did not to impair the spatial learning abilities of the animals (*p* < 0.05, Friedman procedure). During the first trial, a reduction of 51, 47 or 60% in escape latency (*p* = 0.089) was observed after an explosion of 2, 20 or 200 mg/kg compared to controls, respectively (Supplementary Table S2). Such differences between these groups disappeared during the following trials. During the 6th (“probe”) trial, mice exposed to B[a]P at 2, 20 or 200 mg/kg spent more time in the part of the maze where the platform was located compared to controls (Figure 8A) and did a higher number of crossings of this part of the maze (*p* < 0.05, MW-U test modified for multiple comparisons, Figure 8B), suggesting difficulties to adapt to the new situation in these animals.

## 4. Discussion

The present study confirms that B[a]P is a potent toxicant for the brain after oral administration on top of its carcinogenic properties. Additionally, the entero-hepatic metabolism of B[a]P plays an important role in determining the dose necessary to induce neurotoxic effects. The percentages of metabolites recovered in serum 3h after the last administration were significantly higher in mice administered orally (from 9% to 84%—Figure 2A) than those previously intraperitoneally injected with an equivalent dose of B[a]P (from 0% to 59%) [6]. All these non-reactive metabolites showed strong linear relationships between the concentration levels measured in serum and brain. Particular attention can be drawn to the 9-OH-B[a]P and 7,8-diol-B[a]P-metabolites which pointed out the strongest correlation between serum and brain and that were detected in the serum from 2 mg/kg upward, corresponding to the level where behavioural phenotype changes were observed. Both metabolites were also detected in the brain at the lowest dose of 0.2 mg/kg, leading us to suppose that they can either pass the blood–brain barrier and accumulate in cerebral tissue or be produced by local B[a]P metabolism. B[a]P metabolism starts with monooxygenation by microsomal NADPH-dependent cytochrome P450 isoforms 1A/1B (Cyp1A1/Cyp1B1). During phase 1, B[a]P is catalysed by Cyps which oxidised the latter to a B[a]P-7,8-epoxide [43,44]. This intermediary form can subsequently taken over by epoxide hydrolase to form B[a]P-7,8-dihydrodiol. B[a]P-7,8-dihydrodiol can in turn (i) be conjugated, during phase 2, with glutathione, glucuronide or sulfate [45,46,47] or (ii) be a second time oxidised by Cyps to form B[a]P-7,8-dihydrodiol-9,10-epoxide (BPDE). An electrophile attack of this ultimate reactive species at the levels N_2_-Guanine or N_6_-Adenine residues of DNA leads to N_2_-Guanine-BPDE or N_6_-Adenine-BPDE adduct formation. The monohydroxylated forms (1-OH-, 3-OH- and 9-OH-B[a]P) are mostly non-toxic, become even more hydrophilic following conjugation [46] and therefore easily excreted in urine [48]. Diol epoxyde isomers which are not eliminated by this mechanism may generate in addition to DNA adducts, reactive oxygen species in charge of appearance of oxidative stress, known to be involved in the aetiology and development of several neurodegenerative disorders, including Alzheimer’s and Parkinson’s diseases [49,50] and here possibly in the cognitive impairments observed in B[a]P exposed mice. Due to the key role of 7,8-diol-B[a]P in B[a]P metabolic pathway and the strong relationship observed between the measurement of both metabolite in serum and brain, these results raise the question of the suitability of using the measure of both metabolites in serum as a biological indicator of cognitive impairments triggered by B[a]P exposure. Here, B[a]P induced significant overexpression of Cyp1a1/Cyp1b1 at doses of 20 and 200 mg/kg in the 3 brain regions considered, suggesting the ability of the brain to metabolize B[a]P itself [18]. Nevertheless, this change did not correlate with an increasing rate of metabolite production as reflected by the recovery percentages of B[a]P and OH-B[a]Ps in the brain, which remained close to 50% from doses of 2 to 200 mg/kg. A similar recovery pattern was observed for 7,8-diol-B[a]P, with a lower recovery percentage at low doses (9% at 0.2 and 5% at 2 mg/kg) followed by a stable rate around 3% at the 2 highest doses. These results led us to assume either that Cyp1a1/Cyp1b1 activities were saturated at exposure levels above 2 mg/kg, and/or that B[a]P and its hydroxylated-metabolites were transferred through BBB in different proportions according to the dose and way of exposure. Concomitantly, 11 days of oral B[a]P exposure led to an impregnation of B[a]P and its hydroxylated-metabolites in regions of the brain associated with cognitive functions.

Furthermore, exposure induced changes in Nr1 and Nr2a/Nr2b subunit expression in a dose-dependent manner, consequentially modifying the molecular structure of the Nmda receptor and its functionality [51]. Due to the strong implication of the Nmda receptor in the synaptic plasticity in hippocampus and the learning and memory abilities [52], such modifications induced by B[a]P are able to contribute to the behavioural differences related to anxiety and memory presently observed in the same animals. Similar disturbances in *Nr1, Nr2a* and *Nr2b* expression levels were also previously reported in the hippocampus, cerebellum and temporal cortex of mice i.p. exposed to B[a]P with higher proportions in disturbances compared to the present study. This could be the result of the entero-hepatic metabolism contribution in the detoxification of B[a]P orally administered [5,6]. Although *Nr1* expression only increased at doses of 20 and 200 mg/kg in the hippocampus here, it was largely overexpressed irrespective of the dose in cerebellum, hippocampus and hypothalamus in mice i.p. injected [5,6]. Interestingly, *Nr2a* was underexpressed in the temporal cortex at the same doses (20 and 200 mg/kg/day) in both studies. This suggests that i.p. administration of B[a]P can impact the expression of *Nr1* and *Nr2a/2b* subunits, which are required for the synthesis of functional ionotropic *Nmda* receptors [53] and involved in the regulation of the *Nmda* receptor functionality [54], respectively. In contrast, the effects of orally administered B[a]P are limited to the overexpression of such subunits, suggesting the ability of B[a]P to induce more subtle changes in the molecular structure of the *Nmda* receptor when orally administered relatively to the metabolic characteristics of this administration route. As such, our data suggests that oral exposure to B[a]P affects the functionality of the receptor rather than its constitutive organization. This modulation most probably reflects the different biological activity of the altered metabolite profiles.

Our behavioural data shows that oral administration of B[a]P clearly affects the anxiety level while interfering with learning and memory at doses ranging between 2 and 200 mg/kg/day, corresponding to the doses for which both the 9-OH- and 7,8-diol- metabolites can be measured in serum. Taken together, the results of the EPM and the hole board test suggest that B[a]P oral administration at doses higher than 2 mg/kg decreases the anxiety level. We previously reported similar results after i.p. administration of identical B[a]P doses (20 and 200 mg/kg/day) [6], although i.p. administration significantly increased exploration time in the open part of the EPM, which was not the case here. It is noteworthy that B[a]P-treated mice from 2 to 200 mg/kg required less time than the controls and the 2 other B[a]P-treated groups during the 1st trial of the Morris water maze. This may be due to a higher activity level during maze exploration, when the mice were first exposed to the task. This is corroborated by the slightly increased number of holes visited in the hole board test (total and peripheral board) which indicates a concomitant reduction in anxiety level and increase in the exploratory activity.

Otherwise, our results from the Y maze and the learning phase of the MWM during the 5 first trials show the inability of B[a]P to impair the learning performances of the animals whereas rats orally exposed to 2 mg/kg/day of B[a]P for 7 weeks were shown to require more time than controls to explore the maze and found the platform in each of the first 5 trials [55]. This discrepancy may originate in exposure duration, which was considerably longer [55] and could thus lead to higher B[a]P/metabolite levels accumulating in the brain with a need for a longer time to properly locate the hidden platform as a consequence.

In the animals treated with B[a]P from 2 to 200 mg/kg, impairments in working memory performances were observed during the 6th “probe trial” as reflected by the increases in the number of crossings and the time spent in the quadrant where the platform was located. Such results indicate difficulties in adapting to novelty and exploring the maze to find a new potent location of the platform rather than a long-term memory deficit. This result is in line with the modification in the decision-making processes observed in the central part of the EPM in mice exposed at the highest dose of B[a]P. Opposite results were reported by Wang et al. [55] when the hidden platform was removed from the maze, whereas the time needed to reach the location of the platform remained significantly higher in B[a]P-exposed rats compared to controls. Such results suggest a long-term spatial memory deficit rather than an impairment in the specific working memory skills. Since both studies relied on the use of oral gavage as route of exposure, only exposure time can account for the discrepancy observed, suggesting a more acute effect of the treatment here, which could be reversed with extended exposure time. Overall, our results, compared with those of Wang et al. [55], point out the importance of the window of exposure in studying B[a]P-induced cognitive impairment. B[a]P exposure administration route is another determining factor in the severity of the effects observed on learning and memory performances. In this context, we demonstrated in mice administered i.p. with the same doses of B[a]P for 10 days the ability of this PAH to strongly impair the spatial memory and learning abilities in the same task [5]. Oral administration is therefore able to substantially modulate the brain toxicity of B[a]P due to its enterohepatic metabolism in comparison with single hepatic metabolism in the case of i.p. administration.

## 5. Conclusions

This study demonstrated that B[a]P orally administered over a short period of exposure (10 days) causes changes in anxiety and memory-related behaviours and in *Nmda* receptor *Nr1, Nr2a* and *Nr2b* subunit gene expression in different brain areas involved in these cognitive behavioural functions. These results confirm the sensitivity of the glutamatergic Nmda receptor to B[a]P and highlight the role of the entero-hepatic metabolism and the production of particularly reactive metabolites in the sensitivity of the brain to B[a]P when orally administered. The fact that (i) B[a]P is biotransformed for a part into 7,8-diol-B[a]P before becoming a reactive electrophile able to covalently bind DNA and/or to generate reactive oxygen species, (ii) 9-OH-B[a]P and 7,8-diol-B[a]P are detected in serum when behavioural impairments and changes in *Nr1* and *Nr2a/Nr2b* subunit expression *Nmda* are observed, and (iii) there was a strong association between concentration levels of 9-OH-B[a]P and 7,8-diol-B[a]P in serum and those in brain, leads us to assume that both metabolites could be a suitable indicator of cognitive impairment triggered by B[a]P. Further evaluation should be carried out to explore the exact role of each metabolite, especially the 7,8-diol-B[a]P, in the brain toxicity of B[a]P and to confirm the usefulness of the analysis of both metabolites in serum as potent biomarkers of B[a]P-induced cognitive impairments in exposed human populations. 

## Data Availability

Data is contained within the article.

## Supplementary Materials

The following are available online at https://www.mdpi.com/2305-6304/9/3/50/s1, Figure S1: Follow up of body weight of B[a]P-exposed mice *via* oral administration at doses ranging from 0.02 to 200 mg/kg for 10 consecutive days. Results were expressed as mean +/− SEM of 10 mice per group. Table S1: Spontaneous alternation performances of B[a]P-exposed mice *via* oral administration at doses ranging from 0.02 to 200 mg/kg for 10 consecutive days in the Y maze. Results were expressed as median and quartiles (in brackets). Table S2: Performances of B[a]P-exposed mice *via* oral administration at doses ranging from 0.02 to 200 mg/kg for 10 consecutive days in the Morris water maze. Results were expressed as median and quartiles (in brackets) of 10 mice per group.
