# Supplementary material for: Assessment of 9-OH- and 7,8-diol-benzo[a]pyrene in Blood as Potent Markers of Cognitive Impairment Related to benzo[a]pyrene Exposure: An Animal Model Study
Source: Toxics. 2021 Mar 8;9(3):50. doi: 10.3390/toxics9030050 (PMC7998639; doi:10.3390/toxics9030050)
Supplement: Supplementary file 1 [file toxics-09-00050-s001.pdf]

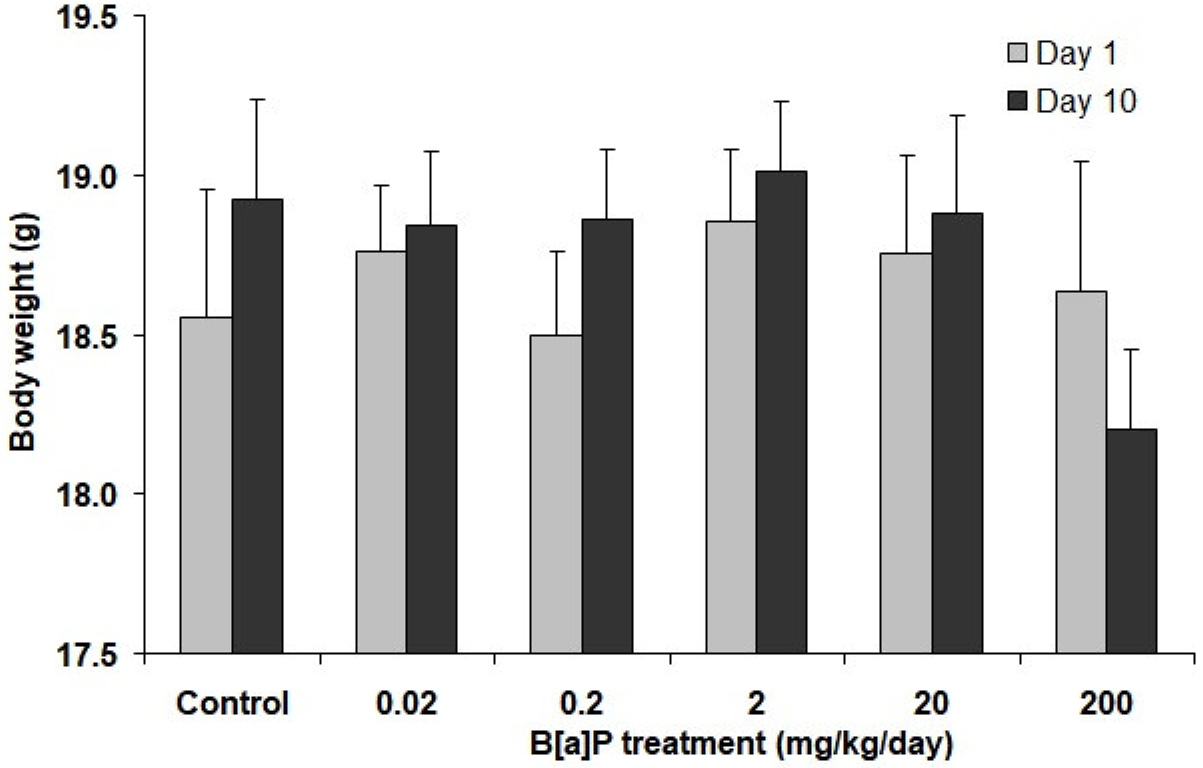

Figure S1. Follow up of body weight of B[a]P-exposed mice *via* oral administration at doses ranging from 0.02 to 200 mg/kg for 10 consecutive days. Results were expressed as mean +/- SEM of 10 mice per group.

Table S1. Spontaneous alternation performances of B[a]P-exposed mice *via* oral administration at doses ranging from 0.02 to 200 mg/kg for 10 consecutive days in the Y maze. Results were expressed as median (first column) and quartiles (in brackets).

MWM	Control	0.02 mg/kg	0.2 mg/kg	2 mg/kg	20 mg/kg	200 mg/kg
% of spontaneous alternation	65 (5–78)	60 (5–72)	66 (5–70)	67 (5–78)	62 (4–79)	62 (4–75)
Total of arm entries	50 (45–58)	52 (41–65)	49 (41–60)	49 (38–60)	49 (42–57)	50 (41–65)
Number of arms visited (min)	5 (5–6)	5 (4–7)	5 (4–6)	5 (4–6)	5 (4–6)	5 (4–7)
Number of arm entries (1st min)	9 (5–10)	8 (6–10)	8 (5–11)	8 (6–11)	7 (1–10)	9 (3–14)

Table S2. Performances of B[a]P-exposed mice *via* oral administration at doses ranging from 0.02 to 200 mg/kg for 10 consecutive days in the Morris water maze (MWM). Results were expressed as median (first column) and quartiles (in brackets) of 10 mice per group.

Treatment	Assay 1	Assay 2	Assay 3	Assay 4	Assay 5
Control	68 (27–92)	37 (14–42)	14 (11–16)	15 (7–17)	36 (19–49)
0.02 mg/kg	59 (11–135)	17 (10–39)	14 (8–17)	10 (8–38)	19 (14–67)
0.2 mg/kg	41 (28–86)	23 (17–26)	14 (10–30)	30 (5–94)	13 (9–64)
2 mg/kg	16 (9–27)	11 (9–22)	23 (9–29)	10 (8–18)	19 (9–31)
20 mg/kg	18 (10–59)	16 (6–22)	21 (7–67)	8 (5–10)	6 (4–7)
200 mg/kg	20 (12–25)	11 (7–23)	17 (6–41)	20 (12–27)	19 (9–55)
